# Vulnerability and Impact Analysis of the IEC 61850 GOOSE Protocol in the Smart Grid

**DOI:** 10.3390/s21041554

**Published:** 2021-02-23

**Authors:** Haftu Tasew Reda, Biplob Ray, Pejman Peidaee, Adnan Anwar, Abdun Mahmood, Akhtar Kalam, Nahina Islam

**Affiliations:** 1 Department of Computer Science and IT, La Trobe University, Plenty Rd., Bundoora 3086, Australia; h.reda@latrobe.edu.au (H.T.R.); a.mahmood@latrobe.edu.au (A.M.); 2 Centre for Intelligent Systems (CIS), School of Engineering and Technology, CQUniversity, Rockhampton 4700, Australia; b.ray@cqu.edu.au; 3 Department of Electrical and Electronics Engineering, Victoria University, Ballarat Rd., Footscray 3011, Australia; pejman.peidaee@vu.edu.au (P.P.); akhtar.kalam@vu.edu.au (A.K.); 4 School of IT, Deakin University, 75 Pigdons Rd, Waurn Ponds 3216, Australia; adnan.anwar@deakin.edu.au

**Keywords:** smart grid, cybersecurity, substation protection, IEC 61850, GOOSE protocol, publish-subscribe communication

## Abstract

IEC 61850 is one of the most prominent communication standards adopted by the smart grid community due to its high scalability, multi-vendor interoperability, and support for several input/output devices. Generic Object-Oriented Substation Events (GOOSE), which is a widely used communication protocol defined in IEC 61850, provides reliable and fast transmission of events for the electrical substation system. This paper investigates the security vulnerabilities of this protocol and analyzes the potential impact on the smart grid by rigorously analyzing the security of the GOOSE protocol using an automated process and identifying vulnerabilities in the context of smart grid communication. The vulnerabilities are tested using a real-time simulation and industry standard hardware-in-the-loop emulation. An in-depth experimental analysis is performed to demonstrate and verify the security weakness of the GOOSE publish-subscribe protocol towards the substation protection within the smart grid setup. It is observed that an adversary who might have familiarity with the substation network architecture can create falsified attack scenarios that can affect the physical operation of the power system. Extensive experiments using the real-time testbed validate the theoretical analysis, and the obtained experimental results prove that the GOOSE-based IEC 61850 compliant substation system is vulnerable to attacks from malicious intruders.

## 1. Introduction

The smart grid has transformed the centrally controlled power system to a fast and massively connected cyber-physical system. While the synergy of a vast number of cyber-physical entities has allowed the smart grid to be much more effective and sustainable in meeting the growing global energy challenges, it has also brought with it a large number of vulnerabilities resulting in breaches of data integrity, confidentiality, and availability. Moreover, it affects a range of unintentional technical issues [1,2] and security vulnerabilities [3,4]. As a consequence, it is critical to analyze the vulnerabilities of the smart grid and identify mitigation techniques [3,5,6]. According to the National Institute of Standards and Technology (NIST) [7], failing to analyze cybersecurity vulnerabilities can lead to the compromise and improper functioning of the physical power system. A cyber criminal or a threat actor can exploit these vulnerabilities, which may lead to malfunctions in energy systems [8], operational failures in both communications equipment, as well as physical d vices, and may even trigger a cascading failure.

To ensure the protection of the smart grid Substation Automation System (SAS) and for reliable message transfer, various communication protocols are being used, which include Modbus/Modbus Plus, Distributed Network Protocol 3 (DNP3), IEC 60870, IEC 61850, and IEEE C37.118. Owing to its provision for security, high scalability, multi-vendor interoperability, and support for various Input/Output (I/O) devices, IEC 61850 [9,10,11,12,13,14] has become the de facto standard for substation communication within the smart grid. The Generic Object Oriented Substation Events (GOOSE) is one of the key communication protocols defined in IEC 61850, which provides a reliable and fast transmission of data and commands between intelligent devices within a substation and between substations.

The recent state-of-the-art works have identified some limitations of IEC 61850. For example, an insecure IEC 61850-based communication infrastructure may allow attackers to gain access, establish communications, and retain a constant presence within a network [15]. To overcome this limitation and provide cybersecurity objectives even across the other communication protocols, the IEC 62351 [4,16] standard was developed. Typically, IEC 62351 Part 6 [16] is the security standard for the IEC 61850 protocol (namely, GOOSE, Sampled Value (SV), and Manufacturing Message Specification (MMS) specifications. However, due to the end-to-end latency requirements of the GOOSE protocol and the constrained computation capabilities of Intelligent Electronic Devices (IEDs) in a smart grid, it is often not feasible to apply the required security measures. For instance, within an SAS, the IEC 61850 Edition 2 communication standard requires that the end-to-end timing requirement of the GOOSE message publication and subscription be within 4 ms considering a 60 Hz frequency power system for a trip command [17], which cannot be guaranteed when high-end encryption is implemented on top of the existing IEDs. For this reason, several IEC 61850-compliant vendors have not yet implemented encryption over their IEDs as the overhead of the encryption algorithm might already incur more than the maximum threshold of the end-to-end IED latency. Consequently, many implementations of the GOOSE protocol remain vulnerable to attacks from intelligent attackers from within and outside the network [12].

Part 6 of the IEC 62351 security standard recommends the use of the asymmetric cryptography-based Hash Message Authentication Code (HMAC) scheme using the RSA digital signature to ensure the basic security requirements of the GOOSE protocol. However, the study in this paper demonstrates the vulnerability of the GOOSE protocol with and without the HMAC for authentication and SHA256 for hash function. Finally, the impact of the cyberattack on the smart grid is demonstrated through analytical approaches and experiments based on real-time simulation.

### 1.1. Contribution

This paper investigates the vulnerabilities of the IEC 61850 GOOSE protocol both with and without the IEC 62351 Part 6 security standard. The investigation analyzes how an adversary can compromise the operation of the power grid by identifying and exploiting the flaws of IEC 61850. Specifically, this paper has the following contributions:

1. A rigorous verification is made of the GOOSE protocol using both automatic and manual security analysis using Scyther. There are various automated security claim verification tools whose detailed reviews can be found in [18,19]. Scyther [18,20] has a number of advantages including its effectiveness for different attack cases, support for multiple protocols, its operational semantics of security protocols, etc. Although other security verification techniques can also be used, in this article, the Scyther tool is utilized where a rigorous security verification of the GOOSE protocol is found using both automatic and manual security analysis. In particular, it is found that the secrecy of the user-defined parameters of the GOOSE protocol, e.g., IED settings, flag, and data, are at risk. Furthermore, the protocol’s data flow is constructed using Scyther’s trace patterns (Section 4 and Section 6).

2. The characterization and completeness of roles for identifying the possibilities of masquerading publishers/subscribers in generating an attack are performed. For example, an adversary can pretend to be a publisher and broadcast a GOOSE message to the Supervisory Control and Data Acquisition (SCADA) control center.

3. We develop an experimental testbed using a real-time hardware-in-the-loop (HIL) emulation for the co-simulation of the power system components and the ICT infrastructure, which reflect a realistic smart grid operation. Using the experimental testbed, it is verified that the identified GOOSE parameter settings in Section 4 are indeed vulnerable to falsified attacks, thus highlighting the weakness of the GOOSE protocol in terms of security.

### 1.2. Organization

Section 2 briefly discusses the related work. Section 3 provides some background on the existing communication standards used in the substation systems focusing on the IEC 61850 and GOOSE protocol. Section 4 mathematically defines the security properties evaluated by Scyther in the automatic claim verification process of the GOOSE protocol. The implementation of the proposed experimental framework and data flow is discussed in Section 5. The experimental validation of GOOSE’s vulnerabilities is presented in Section 6 followed by Section 7, where a falsified attack is generated and its impact on energy system demonstrated. Finally, the paper is concluded in Section 8. Table 1 lists the abbreviations used in this article.

## 2. Related Work

In this section, existing literature related to the security issues of IEC 61850 and the GOOSE protocol in particular, the drawbacks of the related literature, and a concise comparison with our proposed scheme are presented.

The deployment of IEC 61850 has been one of the key transformations in the SAS to fulfill some of the requirements by providing the integration of protection, measurement, monitoring, and control applications via a common communication protocol. However, security issues for the substation based on the IEC 61850 communication standard have come under scrutiny. Especially, IEC 61850’s interconnectivity over a LAN or WAN makes the substation system the main target for intelligent cyberattacks [21]. Hussain et al. [4] provided a thorough overview of the security threats, potential cyberattacks, and security requirements for IEC 61850. Further, the major security flaws of IEC 61850 were reported in [22] as the delay of GOOSE messages and emerging cyberattacks due to the interoperability of IEC 61850 (e.g., via the TCP/IP stack). Thus, there is still a need to analyze the effect of the power system operation if the communication protocol is exploited.

IEC 61850 is vulnerable to a number of attacks, including password cracking, Denial of Service (DoS), and eavesdropping [12]. Similarly, the researchers in [23] presented a taxonomy of various cyberattacks including DoS attack, man-in-the-middle attack, replay attack, injection attack, spoofing, and eavesdropping against IEC 61850-based communication protocols. There have been few research papers [12,24,25] that have demonstrated LAN-based cyberattacks against the GOOSE protocol. The authors in [24] showed a Layer 2-based spoof attack against the GOOSE protocol. They conducted network traffic capture for GOOSE frames using Scapy. Kush et al. [25] proposed GOOSE poisoning over a subscribing IED. They demonstrated the attack by exploiting a vulnerability in GOOSE to effectively cause a hijack of the communication channel. In this work, by injecting attack traffic, their implementation resulted in a DoS attack by denying the authorized publisher’s traffic access to the subscriber. Similarly, an experimental testbed of cyberattack against substation protection was implemented in [26].

Although some existing research works, as explained above, demonstrated attacks against the GOOSE protocol, no research so far has shown a comprehensive vulnerability analysis of the GOOSE communication protocol based on both theoretical claims, as well as experimental simulations. Following the recommendation by Part 6 of IEC 62351 to use the HMAC scheme, the authors in [27] implemented the SHA256 authentication mechanism for the GOOSE protocol. Our paper studies the inherent vulnerabilities of the GOOSE communication protocol within a substation environment considering with and without the IEC 62351 security standard. A systematic vulnerability analysis is conducted to fully investigate the exploitation of the GOOSE protocol. In addition, a falsified trip command injection attack against the weaknesses identified demonstrates that GOOSE is vulnerable to certain types of protocol level attacks. Table 2 provides a comparison of our proposed system with previous research. Therefore, we believe that our research can be an essential input for the technical specification of the IEC 62351 security standard.

## 3. Background: Communication Standards for Substation Protection

**Figure 1:**
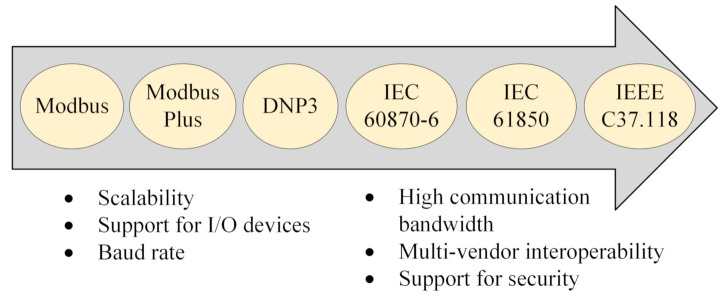
Different communication standards for the substation and SCADA systems.

Various communication standards are being used in the smart grid substation systems including Modbus, Modbus Plus, DNP3 (based on IEC 60870-5), IEC 61850, and IEEE C37.118. Reports [31,32] show that chronologically, Modbus has been used since the late 1970s, and DNP3 has been used since the 1990s. While IEC 60870-6 was introduced in 2000 [32] and has been in use since then, IEC 61850 and IEEE C37.118 are relatively the latest of all communications standards [4]. These communication standards differ from one another in a number of factors, including protocol profile, communication medium (serial, Ethernet, WAN), mode of communication (client-server, peer-to-peer, publish-subscribe, unicast, multicast), communication bandwidth, multi-vendor inter-operability, support for security, etc. [10,14,31,32]. When moving from the older communication standard to the latest one, several communication criteria are improved, such as communication bandwidth, baud rate, scalability, support for several analog/digital devices, multi-vendor inter-operability, support for security, etc. (see Figure 1).

For SAS, IEC 61850 [9,10,11,12,13,14] has become the preferred communication standard because of its improved functionality and several communication requirements including scalability, high bandwidth, multi-vendor interoperability, and security support [4,6,11,22], which also motivated us to further assess the security vulnerabilities of IEC 61850-based protocols.

### 3.1. IEC 61850

IEC 61850 offers a wide range of data modeling, which is based on self-describing information in a system independent of the vendor (i.e., interoperability). The communication protocols under IEC 61850 support client-server and publish-subscribe communication models. The IEC 61850 communication standard supports the following communication protocols: Generic Substation Status Event (defined in IEC 61850 Part 7-2), client-server model-based MMS (defined in IEC 61850 Part 8-1), SV (defined in IEC 61850 Part 9-2), and GOOSE (defined in IEC 61850 Part 8-1). The SV (used for the transfer of sampled values) and GOOSE (used for substation events) protocols are utilized for the transmission/reception of data in time-critical functions based on a publish-subscribe communication model. Data transmission/reception in publish-subscribe communication is carried out asynchronously (in contrast to a synchronization approach in a client-server communication model) and through multicast communication (i.e., one-to-many and many-to-many). The multicast communication allows the publisher to send just a single copy of data to the network, which is then transmitted to all subscribers that have already shown their interest. This will increase the overall performance of data delivery and minimize end-to-end latency and traffic over the network.

### 3.2. GOOSE Protocol

In general, GOOSE messages can be transmitted over a LAN (i.e., for applications within the substation), or routable GOOSE messages can be multicast over a WAN for inter-substation applications [33]. This paper focuses on the GOOSE protocol that consists of the three Open Systems Interconnection layers: physical layer, data link layer, and the application layer, as defined in IEC 61850 Part 8-1 [10]. It is mainly used for event-based and time-critical data transfer between unit-level devices (such as a relay) and process-level devices (such as a circuit breaker), or at the substation level. Each network entity (such as the IED) that is on the publish-subscribe network communicates with others by sending data to the shared LAN and receives data asynchronously.

Within a substation, a publishing IED (PubIED) generates a GOOSE message and sends it to the network via multicast transmission over full-duplex-based Fast Ethernet and a high-speed switch network. Each PubIED should know all the subscribers to whom to send the GOOSE data and even the other PubIEDs. Similarly, any IED that wants to receive a GOOSE message should first subscribe to the network as each subscribing IED (SubIED) should know the list of all publishers and all other SubIEDs to update their list of potential publishers or their future subscribers. The main objective is to ensure that the PubIED is capable of sending GOOSE data when a deviation in an event occurs in the substation system, and that SubIED(s) subscribes to the transmitted data.

## 4. Definition of the Security Properties Evaluated by Scyther 

This section discusses in detail the tools and technique used for the vulnerability analysis of the IEC 61850-based GOOSE communication protocol in the publish-subscribe scenario. IEC 62351 [16] is a de facto security standard for IEC 61850, and thus, the communication flow of GOOSE as applied to the data flow between the protective/controlling IEDs has been considered both with and without the compliance of IEC 62351. Due to the rapid development and evolving nature of technologies and their associated communication protocols, a large number of security protocols have been developed and deployed in order to provide secure communication [8]. The analysis of security protocols is becoming increasingly difficult for manual and analytical techniques like ’Gong–Needham–Yahalom’ (also called the GNY) logic and ’Burrows-Abadi-Needham’ (also called the BAN) logic [18]. Therefore, the formal analysis of the security protocol relies more on an automated falsification or verification of such protocols using state-of-the-art tools such as the ProVerif or AVISPA [34] tools, which have been shown to be effective at finding attacks on protocols (AVISPA) or establishing the correctness of protocols (ProVerif). One of the improved and automated security claim verification tools used in the literature is Scyther [18,19,35,36], which is based on a pattern refinement algorithm, which can provide concise representations of (infinite) sets of attack traces. In this experiment, Scyther is used to perform the security analysis of the GOOSE communication protocol. Using the Scyther tool, the GOOSE protocol is analyzed in three main areas:

- to verify whether the security claims in the protocol description hold or not;
- to automatically generate appropriate security claims for a protocol and verify these;
- to analyze the protocol by performing complete characterization.

In contrast to individual traces, Scyther works based on reasoning about classes of traces by representing them using trace patterns, which is defined as a partially ordered and symbolic set of events. In this paper, various Scyther definitions are considered like trace pattern, complete characterization, algorithm, bound on runs, and completeness for verification of the security properties in Section 6, as defined below in Definitions 1, 2, 3, and 4, respectively. These definitions are adapted from [37] and used in Section 6 for the verification of the security properties of the protocol by analyzing patterns.

**Definition** **1** (Trace pattern)**.** *Let P be a protocol, and let TP be a trace pattern. Let ES be a set of explicit trace patterns. The set of traces of the protocol that exhibit the pattern is defined as:*

(1) traces(P,(TPE,→))=traces(P)∩traces(TPE,→)

**Example** **1.** *Let*P*be a protocol with two events*E1, E2*such that*E1≺${}_{r}$E2. *Let*e1=〈inst〉E1*and*e2=〈inst〉E2*for some instantiating function, then we have Equation (2) below.*

(2) traces(P,({e1,e2},{e2→e1}))=∅

This pattern does not occur in the traces of the protocol, because according to the operational semantics, the events of a single run of any protocol should conform to the protocol role order.

**Definition** **2** (Complete characterization)**.** *Let P be a protocol, and let TP be a trace pattern. Let ES be a set of explicit trace patterns. ES is called a complete characterization of the set traces (P,TP), if and only if Equation (3) is true.*

(3) $$\underset{E\in ES}{⋃}traces\left(P,E\right)=traces\left(P,\mathrm{TP}\right)$$

**Definition** **3** (Characterization algorithm)**.** *Given an iterative protocol (like GOOSE) with the signature as Equation (4):*

(4) Algorithm(ψ):Protocol×Pattern→P(Pattern)

*Given a trace pattern TP and a protocol P, the algorithm yields a set of explicit trace patterns that represent a complete characterization of the protocol as Equation (5).*

(5) $$\underset{E\in \psi (P,TP)}{⋃}traces\left(P,E\right)=traces\left(P,\mathrm{TP}\right)$$

**Definition** **4** (Bound on the number of runs and completeness)**.** *For all protocols P, trace patterns (TPE, →) bound on the number of runs as maximum runs (maxr) and sets of explicit trace patterns ES, where F stands for false, we have Equation (6).*

(6) $$\begin{array}{c} (\psi^{maxr}\left(P,\left(\mathrm{TPE},\to \right)\right)=\left(\mathrm{ES},\left(F,\to \right)\right)⟶\psi \left(P,\left(\mathrm{TPE},\to \right)\right)=\mathrm{ES} \end{array}$$

As derived in Equation (6), after termination of the bounded version of the algorithm, no patterns are deemed contradictory on the basis of having surpassed the maximum number of runs; the resulting realizable trace patterns represent a complete characterization of the trace pattern of the protocol. The Scyther simulation tool is built on the principle and definition presented in Equation (6). Upon initial testing, the Scyther processes the SPDL code of the protocol to ensure the compliance of the runs and the completeness of the protocol’s process. Therefore, the Scyther simulation captures all possible behaviors of the trace pattern.

The experimental validation of the proposed assessment technique is illustrated in Section 6.

## 5. Implementation

In this section, the laboratory setup of the substation protection system is discussed including the equipment used, the configuration of the IEDs, as well as the architecture and data flows of the communication network. Next, the experimental validation of the claims proposed in Section 4 is explained. Finally, it is demonstrated with experimental results how the injection of falsified information in the GOOSE protocol can cause operational disorder in the physical power grid.

### 5.1. Proposed Cyber-Physical Testbed

**Figure 2:**
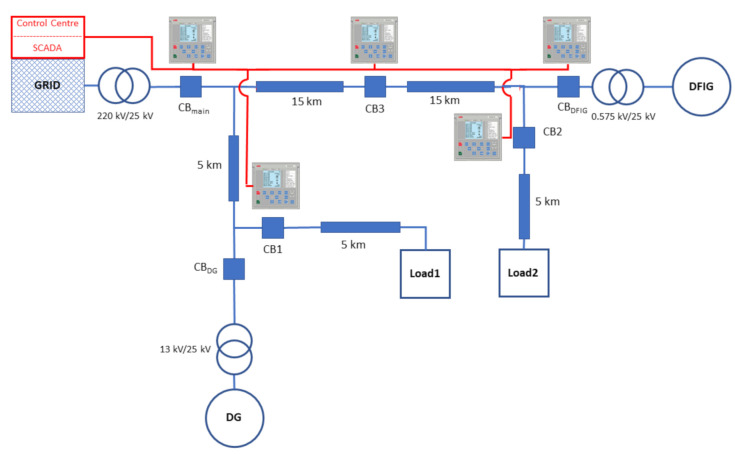
Architecture of the proposed test system.

Figure 2 shows the overall system architecture of the physical model of the power system, which represents a typical microgrid consisting of an emergency Diesel Generator (DG), a Double-Fed Induction Generator (DFIG) wind farm, and two load centers. During grid-connected mode, the microgrid is connected to the main grid through a 220 kV/25 kV step-down transformer and a mains circuit breaker ($CB_{main}$) enabling both grid-connected and islanded modes of operation.

To develop the experimental testbed, a real-time co-simulation of the power system components and ICT infrastructures consisting of communication network elements and protection IEDs is adopted. Thus, to establish a real-world scenario for the interaction between power system elements and IEDs within the power protection system, the concept of HIL using OPAL-RT is adopted to ensure real-time characteristics for the experimental testbed. The OPAL-RT (OPAL-RT provides the hardware PC/FPGA-based real-time simulators for testing equipment and Rapid Control Prototyping (RCP) systems. Details can be found at https://www.opal-rt.com/ (accessed on 6 January 2021)) platform offers real-time simulations and HIL testing facilities for the design, testing, and optimization of physical systems used in a number of industries, including the power grid for industrial use, as well as academic studies. To simulate the power system operations of our proposed architecture (Figure 2), a high-performance real-time digital simulator known as OP5600 (OP5600 is a high performance equipment for real-time simulations of physical systems for research and industry applications. Details can be found at https://www.opal-rt.com/simulator-platform-op5600/ (accessed on 6 January 2021) is used at the Victoria University Zone Substation (VUZS) laboratory [38,39]. Moreover, the OP5600 chassis supports the IEC 61850 communication standard. Additionally, two work stations are used as clients to run the IEC 61850-based GOOSE protocol.

**Figure 3:**
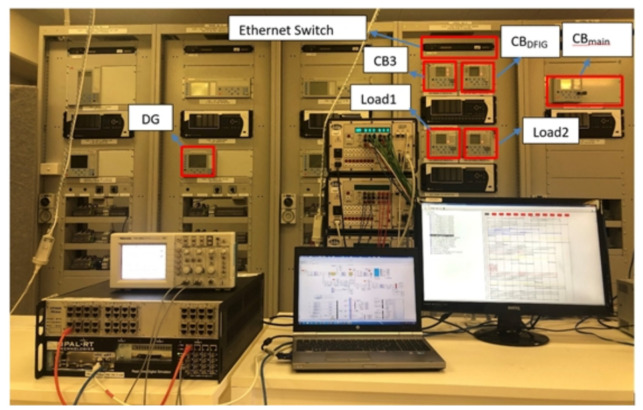
Practical setup of the proposed test system.

In parallel with the power system simulation, the power protection system is developed over a LAN where IEDs from ABB (namely the REF615 (REF615 is a dedicated feeder IED for protection and control applications from ABB which is compatible with the IEC 61850 communications standard. Details can be found at https://new.abb.com~/products/REPREF615/ref615 (accessed on 6 January 2021)) relays) are configured to communicate their data using the IEC 61850 GOOSE messaging service. The data communication between the real-time simulator and the IEDs in the power protection system is achieved through a LAN-based network interface card interface on the OPAL-RT chassis where a LAN-based switch is also utilized. From Figure 2, the LAN for data communication between IEDs is represented by the red dashed line. Figure 3 shows the actual laboratory setup, which includes the CBs and their respective IEDs. Table 3 summarizes the cyber-physical equipment, tools, and methods used in this experiment.

### 5.2. Data Flow for Communication

Here, the communication flow for the proposed system is demonstrated. During the experiment, a simulated GOOSE message is used based on OPAL-RT’s real-time simulation environment. The exchanged data include issuing tripping commands (opening or closing CBs), interlocking functions, and reading status. The data flow of the GOOSE protocol in the proposed system has the following steps.

**Figure 4:**
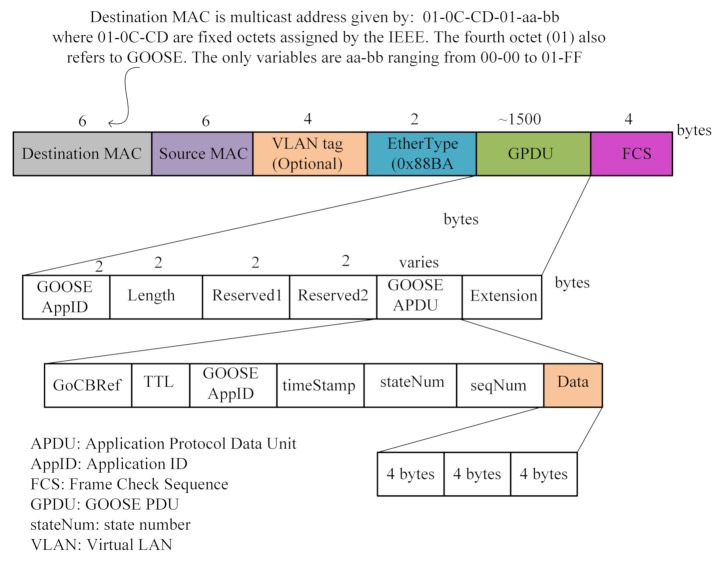
GOOSE frame structure.

***Step 1***: In this step, user-defined data are generated. During IED configuration, four kinds of IED settings are defined (see the GOOSE frame structure in Figure 4). Each setting designates a state or a function of the protective relay and is represented by three bits. These data are shown in Figure 4 by the *Data* field. As four SubIEDs are considered, twelve bits are transmitted to the multicast network by the PubIED.

***Step 2***: Here, the generated data are sent to the PubIED.

***Step 3***: The PubIED encodes, maps, and models the data compliant with the IEC 61850 data modeling [13].

***Step 4***: After the encoding and data modeling in Step 3, the PubIED generates the GOOSE message. The generated GOOSE data are mapped to the Ethernet data frame, eliminating headers of the other higher layers of the Open Systems Interconnection model. In this paper, the GOOSE data frame structure is analyzed following the definitions of the GOOSE protocol in IEC 61850 Part 8-1 [10,13].

***Step 5***: The GOOSE frame is published to the LAN-based multicast network.

***Step 6***: Here, every subscribing IED will receive the message and filter out the message if the IED’s settings match the received GOOSE message.

For the protection application in a smart grid substation system, the IEC 61850 Edition 2 communication standard requires that the end-to-end GOOSE data transfer should be within 4 ms considering a 60 Hz frequency of the power system for one of the following message types: *trip*, *interlocking*, *inter-trips*, and *logic discrimination between protection functions* [17].

The data transfer time includes the time taken by the PubIED when encoding, processing, and mapping the generated user signal into the GOOSE dataset, when transmitting the GOOSE data to the network, and when this GOOSE message is being received and processed by the SubIEDs. Under the GOOSE model, there is no acknowledgment between the SubIED and PubIED. Hence, the PubIED ensures the reliability of data delivery to the SubIED by means of multiple retransmissions, with a variable and decreasing step until a stable condition is reached as follows. Each GOOSE message in the retransmission sequence carries a TTL parameter to inform each SubIED of the maximum time to wait for the next re-transmission. The retransmission time is stopped when a new event occurs in the substation with an indication of a new incoming GOOSE frame. The state number (stateNum) within the GOOSE frame structure identifies whether the GOOSE frame is a new message or a retransmitted one.

**Figure 5:**
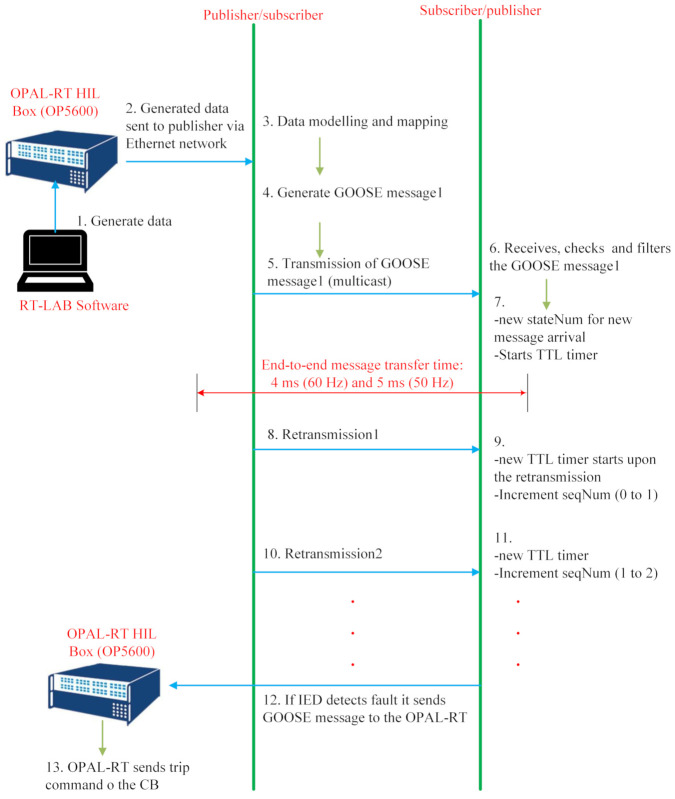
Data flow of the GOOSE protocol without IEC 62351.

The data flow of the GOOSE protocol is illustrated in Figure 5 and summarized below:

1. From the PubIED side:

- The PubIED sends a new GOOSE message to the network. The retransmission timer is started based on the PubIED’s TTL parameter. Sequence number (seqNum) is set to zero. It is recommended that the retransmission timer be less than (actually half) of the TTL value.
- The retransmission time expiration indicates the seqNum is incremented.
- Upon retransmission, a GOOSE message is transmitted, and the next retransmission interval is used.

2. From the SubIED(s) side:

- SubIED(s) receives a new GOOSE message, then begins a TTL timer.
- The TTL timer expires.
- It will receive another retransmission or a new GOOSE message.

When an IED detects a fault, it sends a GOOSE message to the OPAL-RT (***Step 12***). Then, the OPAL-RT will send a trip command to the CB (***Step 13***).

## 6. Experimental Validation of GOOSE’s Vulnerabilities

The aim of this experiment is to identify the possible vulnerabilities and attack trace patterns of user-defined parameters and roles in an insider attack scenarios of the GOOSE message exchange protocol. The experiment uses trace patterns to verify the complete characterization and correctness of the roles involved in the communication as defined in Section 4. This is important in falsified insider attack scenarios as any roles can act as a malicious intruder.

### 6.1. Experimental Setup

**Figure 6:**
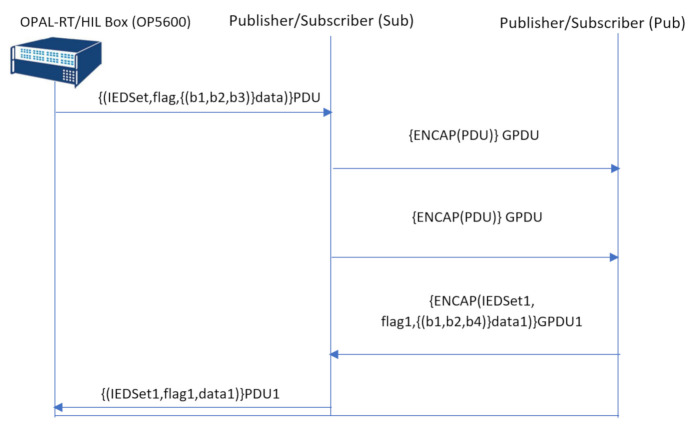
SPDL flow of the GOOSE protocol without IEC 62351.

The GOOSE protocol description was written in the Security Protocol Description Language (SPDL) [18] semantics to interpret the communication flow of the protocol in a Scyther understandable format. As illustrated in Figure 6, the GOOSE communication flow starts from OP5600 by sending three user-defined parameters below, named the Protocol Data Unit (PDU), to the publisher.

- IEDSet: illustrated as the IED settings in Figure 4;
- flag: detailed in Figure 4;
- data: user-defined data in OP5600 as illustrated in Figure 5.

**Figure 7:**
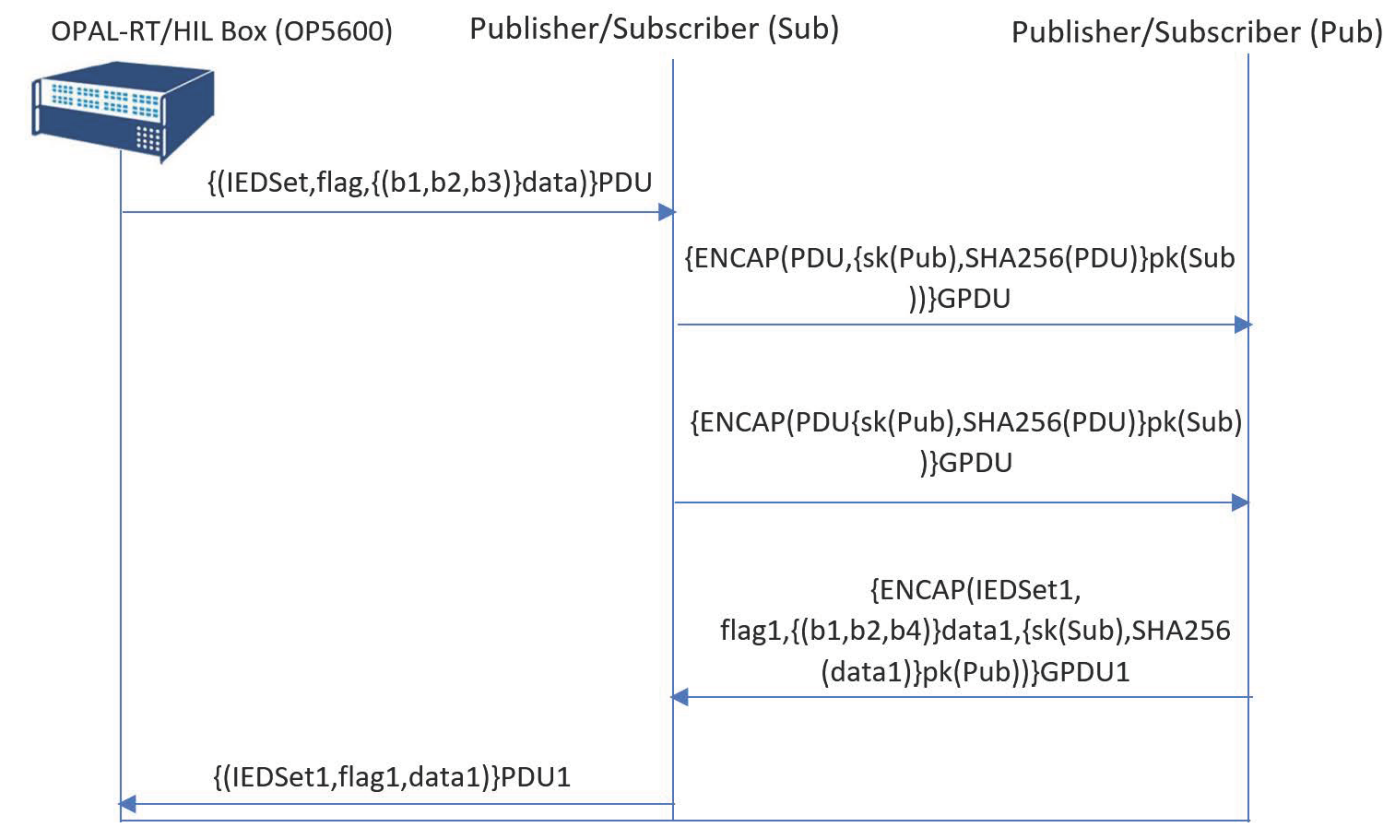
SPDL flow of the GOOSE protocol with IEC 62351.

The publisher prepares the GOOSE message as ENCAP(PDU)GPDU (ENCAP = Encapsulate), which includes all the header information and the PDU, as illustrated in Figure 7. For our security analysis, all header information is presented as ENCAP. Additionally, all header information within the ENCAP are suppressed to make the vulnerability analysis more focused towards the three user-defined parameters in the context of an insider attack scenario. The publisher then broadcasts the GPDU to all subscribers. In the next transmission, the subscriber and publisher interchange their roles and transmit GOOSE messages with the same format, but different user setting values to mimic actual power system scenarios. In the final transmission, the subscriber updates the SCADA system based on the received information, as illustrated in Figure 6.

In Figure 7, the IEC 61850 security extension, namely IEC 62351, is considered with respect to the implementation described in Figure 6. The IEC 62351 security extension uses the HMAC with a digital certificate to verify the authenticity of the GOOSE message. SHA256 is considered for the implementation of the hashing security extension of Scyther.

Along with the SPDL code to interpret both Figure 6 and Figure 7, the settings presented in Table 4 were used to configure Scyther. As shown in Table 4, the protocol ran five interaction and best attack scenarios settings in our simulation. Here, five iterations ensured that the simulation considered trace patterns throughout the five executions of the protocol. The settings of the simulation are adjustable based on the need of the actual system. The proposed method works as an incremental process and a passive plug-in to collect the pattern of live executions from the actual system. Therefore, there will be very little or no impact of the proposed method on the actual system.

Table 5 shows the security claims Alive, Weakagree, Nisynch to prove the correctness and to determine the complete characterization of the roles [35] and [20]. Furthermore, the secrecy of user-defined values (flag, data, and IEDSet) is tested in our experiment. The definition of the Scyther security claims were adapted from [35] and [20].

### 6.2. Results and Discussion

The Scyther experiment on the GOOSE publish-subscribe-based protocol reveals some insight about the vulnerabilities that a smart power system may face from a malicious insiders. The results are presented in two main categories: role characterization and security claim verification in Table 6 and Table 7, respectively. Moreover, the outcome of the automated security claim verification is presented in Table 7.

As is shown in Table 6, there are many trace patterns relevant to a role, whereas in a secure protocol, there must be only one trace pattern to ensure the correctness and completeness of a role [35] and [20]. These excess trace patterns are vulnerabilities that can be exploited to create falsified attacks. It is important to note that although the security extension (implementation of IEC 62351) helped to reduce the number of trace patterns, due to the unencrypted transmissions and multicasting of the same information repeatedly, there is still a significant number of trace patterns that can be used to falsify the user-generated data of GOOSE messages by an insider attacker.

**Figure 8:**
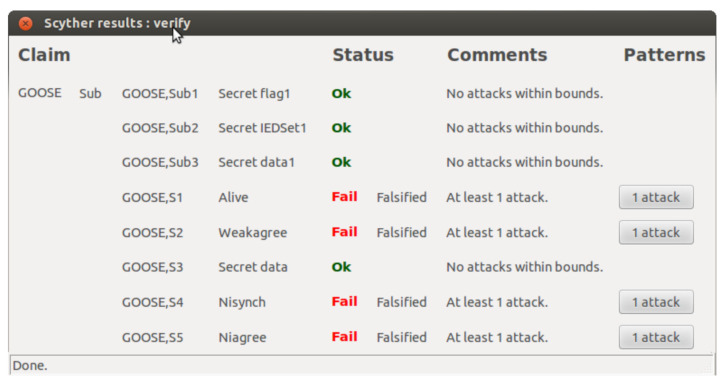
GOOSE security verification using Scyther.

**Figure 9:**
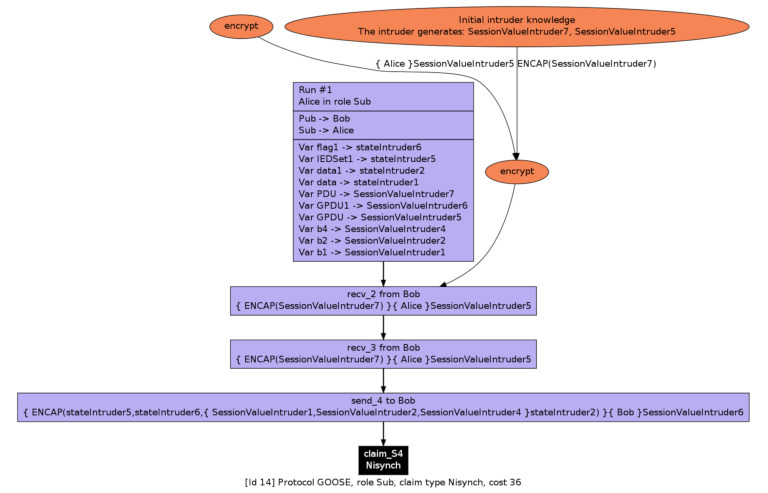
Attack on subscriber to compromise the Nisynch security property.

It can be seen from Table 7 that most of the automated and manual claims related to user data have one attack scenario at the publisher’s end as the publisher has the authority to repeatedly send the same message to a group of subscribers in plain text. At the same time, user data are less prone to falsified attack at the subscriber’s end as it only reads data with a standard set of criteria. Furthermore, it is important to note based on our simulation result that all three roles, namely Alive, Weakagree, Nisynch, and Niagree, failed to comply with the four characterization security properties. This is due to the excessive trace patterns that can be used by inside attackers to exploit all roles involved in the GOOSE message communication. This can further be confirmed by the result in Figure 8. One of such attacks, presented in Figure 9, illustrates that an intruder Alice masquerading as a Sub (Subscriber) can use header information ENCAP by taking advantage of the publisher’s re-transmissions and thereby fool legitimate subscribers to accept malicious PDU and GPDU that have been prepared using the attacker’s user-defined values.

## 7. Falsified Attack Generation and Impact Analysis

In this section, following the vulnerability analysis of the GOOSE protocol discussed earlier in Section 4, an insider cyberattack scenario is demonstrated. In this demonstration, an insider attacker who might have familiarity with the substation network architecture is considered. In particular, a Layer 2 attack based on MAC spoofing is considered. In other words, the attacker clones the MAC address (which is a very simple technique in most operating systems) of the publisher and tries to alter the contents of the GOOSE message.

First of all, from the attacker’s side, a packet sniffing attack is performed using a network traffic analyzer called Wireshark (It is an open-source software for network and communication protocol analysis. Details can be found at https://www.wireshark.org/ (accessed on 6 January 2021)). From the Wireshark packets, the attacker learns some parameters such as the source and destination MAC addresses and the data content of the GOOSE frame. Then, the attacker clones the source MAC address to mimic a publisher. Next, the attacker generates false data, by altering the 12 bit data frame in the user-defined field of the IED settings. Furthermore, the attacker encodes and publishes the modified GOOSE message to the LAN-based multicast network where the subscribing IEDs receive the modified data, even for different re-transmissions. For the experimental setup to encode GOOSE data messages, Eclipse (a Java-based development environment) was used.

**Figure 10:**
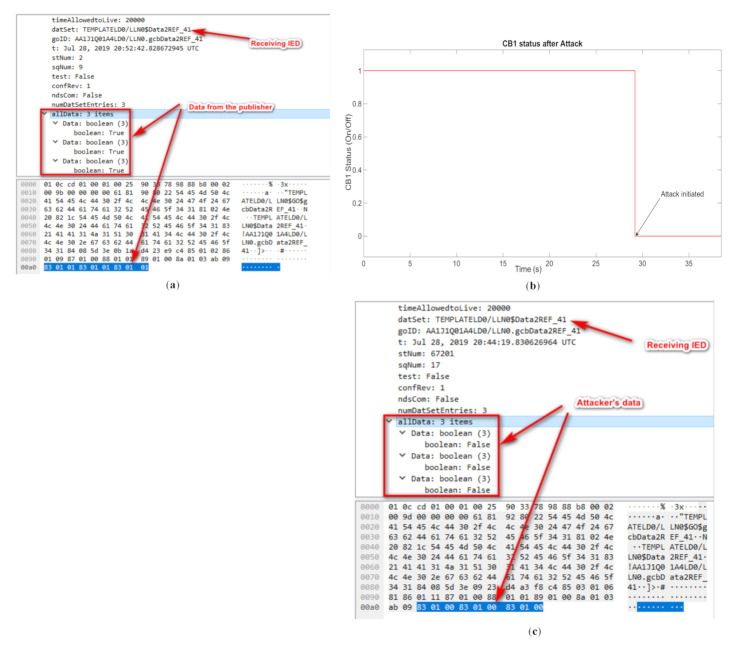
(**a**) GOOSE message captured before attack, (**b**) change in CB1 status and (**c**) GOOSE message captured after attack.

In the implementation phase, the network traffic for both the publisher’s and attacker’s side is monitored. Figure 10a shows a packet captured via Wireshark for the real network traffic transmitted by the PubIED. As is shown in Figure 10a, the publisher sends 111-111-111-111 (all true Boolean values representing the IED settings as explained earlier). Furthermore, from the attacker’s side, the attack scenario is initiated on CB1, where the IED settings are manipulated in the GOOSE data message frame and published to the network. In Figure 10b, the resultant change in CB1’s status from closed (1) before attack to open (zero) after attack is illustrated. The attack is initiated in CB1 around 29.2s later after the start of the simulation, shown in Figure 10b, where the circuit breaker trips due to the falsified command. This is also reflected in the Wireshark packet capture tool, shown in Figure 10c.

**Figure 11:**
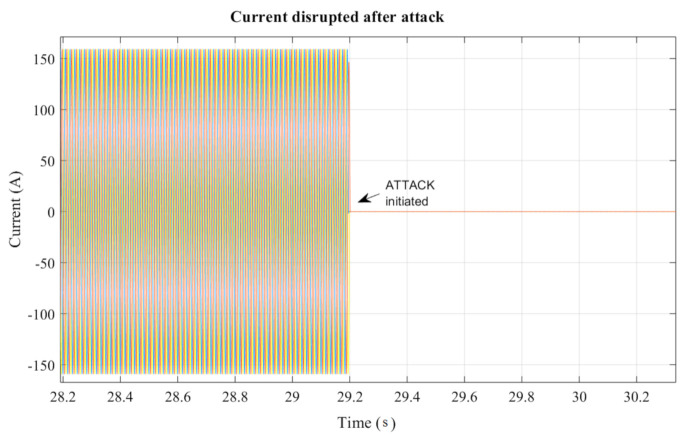
Disruption in the current measurement after injection attack.

**Figure 12:**
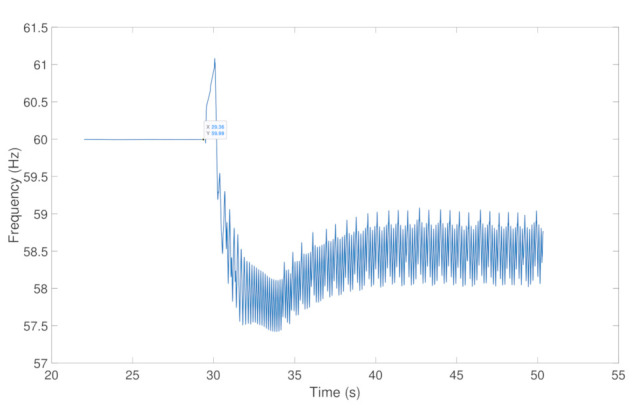
Frequency excursion after injection attack.

Finally, from the perspective of power system operation, the resulting effect of the cyberattack on CB1 is illustrated in Figure 11, where the undesirable operation of CB1 leads to the interruption of the power supply (current) for consumers downstream of the distribution network. It can be observed that no current flows after CB1 is tripped due to the falsified information injected into the GOOSE protocol. Moreover, this can further induce abnormality in the operation of the power system, causing frequency excursion and cascading failures. In fact, Figure 12 highlights the cascaded effect of the undesirable operation of CB1 under the proposed cyberattack scenario, which leads to a large-scale failure of power system operation.

## 8. Conclusions

IEC 61850 is one of the latest communication standards for power grid substation networks. IEC 61850 includes various communication protocols, including the GOOSE protocol. This article assessed the vulnerabilities of the GOOSE protocol, with and without the IEC 62351 security scheme. To validate the proposed architecture, analytical approaches and real-time HIL-based simulations are performed. In particular, the complete characterization of the roles of the protocol are performed, and automatic security claims are verified to showcase the impact of data injection attacks. Both analytical approaches and HIL-based experimental results demonstrate that the GOOSE publish-subscribe-based communication of the smart power grid exhibits security vulnerabilities and is prone to malicious attacks even under the compliance of the IEC 62351 security standard. In the future, the vulnerabilities and impact of emerging data integrity cyber-physical attacks against power system operations across inter-substation networks can be investigated considering the IEEE C37.118 communication standard. In addition to the vulnerability and impact analysis of cyberattacks, a comprehensive cyber-physical security solution is required to deter the investigated security flaws. For example, defense countermeasures for the cyberattacks against the communication protocols of inside substation networks and beyond can be proposed.

## Figures and Tables

**Table 1:** List of abbreviations.

Abbreviation	Description	Abbreviation	Description
CB	Circuit Breaker	NIST	National Institute of Standards and Technology
DNP3	Distributed Network Protocol 3	PDU	Protocol Data Unit
DoS	Denial of Service	Pub	Publishing
GOOSE	Generic Object-Oriented Substation Event	SAS	Substation Automation System
HIL	Hardware-In-the-Loop	SCADA	Supervisory Control and Data Acquisition
HMAC	Hashed Message Authentication Code	SHA	Secure Hash Algorithm
IEC	International Electrotechnical Commission	SPDL	Security Protocol Description Language
IED	Intelligent Electronic Device	Sub	Subscribing
LAN	Local Area Network	SV	Sampled Value
MAC	Media Access Control	TTL	Time To Live
MMS	Manufacturing Message Specification	WAN	Wide Area Network

**Table 2:** Comparison of our proposed system with previous works.

Comparison Attributes	Our Proposed System	Previous Works
Communication flow of the GOOSE protocol	Considered comprehensive publish-subscribe communication flow with experimentation over IEDs	Considered in [4,22,24,28]
HIL-based cyber-physical testbed for IEC 61850 security	Used OPAL RT-based HIL testbed for GOOSE-based IEC 6850 security	Used real-time digital simulator-based testbed environment for the MMS-based IEC 61850 security in [29]; in [30], IEC 61850 security was investigated using the OpenIEC61850-based testbed
Cyberattack scenario and impact analysis	Generated falsified trip command injection based on the HIL experimental testbed and investigated its impact on the substation network	Attack against protection in the substation and the impact considered in [26], GOOSE poisoning against subscribing IEDs in the substation considered in [25], and spoof attack against the GOOSE protocol considered in [24]
Security claim verification for the GOOSE protocol	Considered Scyther for security claim verification	Not considered
IEC 62351 security for the GOOSE protocol	Considered with and without IEC 62351 security using SHA256 for the communication flow and GOOSE vulnerability analysis	Not considered

**Table 3:** Devices, tools, and methods used in the experiment.

Device, Tool, or Method	Description
OP5600	Real-time digital simulator
IEC 61850	IEC 61850 protocol card
REF615	Protective and control relay of power lines
Ethernet switch	LAN connectivity
SPDL	Software tool for security protocol verification
Wireshark	Network traffic analysis for GOOSE message sniffing
Eclipse	Java IDE for encoding GOOSE data messages

**Table 4:** Scyther’s configuration detail for the experiment.

Options	Values
Runs	5
Type	Type matching
Search pruning	Best attack scenarios
Maximum number of patterns per claim	10

**Table 5:** Security claims for the GOOSE protocol’s communication.

Role(s)	Claims	Values
Pub and Sub	Alive	N/A
Pub and Sub	Weakagree	N/A
Pub and Sub	Nisynch	N/A
Pub and Sub	Niagree	N/A
Pub and Sub	Secret	flag
Pub and Sub	Secret	IEDSet
Pub and Sub	Secret	data

**Table 6:** Role characterization for correctness and completeness.

Role(s)	Status	With Security (WS)	No Security (WOS)
OP5600	Reachable(ok)	435 trace patterns	493 trace patterns
Pub	Reachable(ok)	972 trace patterns	1069 trace patterns
Sub	Reachable(ok)	36 trace patterns	37 trace patterns

**Table 7:** Auto and manual security claim verification.

Claims	Role Involved	WS	WOS
Secret data	Pub	1 attack found	1 attack found
Secret data	Sub	No attacks	No attacks
Secret flag	Pub	1 attack found	1 attack found
Secret flag	Sub	No attacks	No attacks
Secret IEDSet	Pub	1 attack found	1 attack found
Secret IEDSet	Sub	No attacks	No attacks

## Data Availability

Data sharing not applicable.
